# Torsional Behavior of WaveOne Gold Endodontic File with the Dedicated Motor of the Original WaveOne File

**DOI:** 10.3390/ma11071150

**Published:** 2018-07-06

**Authors:** Jung-Hong Ha, Asgeir Sigurdsson, Gustavo De-Deus, Antheunis Versluis, Sang Won Kwak, Hyeon-Cheol Kim

**Affiliations:** 1 Department of Conservative Dentistry, School of Dentistry, Kyungpook National University, Daegu 41940, Korea; endoking@knu.ac.kr; 2 Department of Endodontics, New York University College of Dentistry, New York University, New York 10010, NY, USA; asgeir.sigurdsson@nyu.edu; 3 Department of Restorative Dentistry, College of Dentistry, Universidade Federal Fluminense, Rio de Janeiro 24220-900, Brazil; endogus@gmail.com; 4 Department of Bioscience Research, College of Dentistry, University of Tennessee Health Science Center, Memphis, TN 38163, USA; averslui@uthsc.edu; 5 Department of Conservative Dentistry, School of Dentistry, Dental Research Institute, Pusan National University, Geumo-ro 20, Mulgeum, Yangsan 50612, Korea; endokwak@pusan.ac.kr

**Keywords:** distortion angle, reciprocating nickel-titanium file, safety angle, torsional resistance, WaveOne, WaveOne Gold

## Abstract

This study compares the safety limits and torsional resistances of WaveOne Gold (WOG) and WaveOne (WO) endodontic files while using the original motor. The safety limits for reciprocating angles were tested by measuring the torsional limit before plastic deformation (TLP) after repetitive torsional loading at gradually increasing load, and after single continuous rotation. Statistical analysis was performed by independent *t*-test at 95% confidence level. The tested specimens were observed under a scanning electron microscope (SEM). Regardless of the test mode, the distortion angle at the TLP was higher for WOG than WO (*p* < 0.05), and all were greater than the 150-degree rotation of the original motor. The mean values of the distortion angle and torque from the single continuous rotation loading were significantly lower than those with repetitive loading movements. Comparing the two systems under SEM, WO showed a catastrophic change in comparison with WOG. Evaluation of the lateral aspects showed longitudinal micro-cracks after 270-degree repetitive movements. After 150-degree repetitive movements, no distorted machining grooves were found in either group, but WO showed evidence of longitudinal micro-cracks. Under the conditions of this study, the torsional loading from the pre-set mode of the dedicated motor for WO was safe for WOG as well.

## 1. Introduction

Nickel-Titanium (NiTi) rotary instruments are now essential equipment in endodontic treatment, offering high flexibility, enhanced cutting proficiency, and the ability to preserve root canal anatomy compared to stainless steel files [1,2]. Notwithstanding these advantages, the fracture of NiTi instruments is still a major concern for clinicians [3,4]. Fracture modes of rotary NiTi instruments have been categorized as either cyclic flexural fatigue or torsional failure [3]. Mixed mode failures, under the clinical circumstances, can be caused by the simultaneous effect of torsion and flexure stress [5,6,7]. Researchers and manufacturers have tried to improve fracture resistance of instruments as well as clinical efficiency in a variety of ways. Geometric modifications, heat treatments, surface treatments, and altered kinematics have all be used to enhance the mechanical properties and clinical performance of NiTi instruments [7,8,9,10,11,12].

Reciprocal movement, that periodically reverses the rotational direction of instruments, has been proposed with NiTi instruments in an attempt to reduce the torsional stress. Two NiTi instrument systems were introduced that follow this reciprocation concept: Reciproc (VDW, Munich, Germany) and WaveOne (Dentsply Sirona, Ballaigues, Switzerland). Several studies have shown that the use of reciprocating motion can extend the lifespan of a NiTi instrument and thus may increase clinical efficiency compared to continuous rotation [13,14,15].

The next generation of WO and Reciproc, WOG (Dentsply Sirona) and Reciproc Blue (VDW), respectively, are made from differently heat-treated NiTi alloy (gold-wire and blue-wire respectively) than the old WO and Reciproc, which were made from M-wire. Heat-treatment could modify the micro-structure of NiTi instruments and subsequently result in modified physical properties [8,9,12,16,17]. Not only are the new files made from alloys with different properties, but the geometric designs of the WOG are radically modified with a parallelogram-shaped cross-section and smaller tapered tip than the old WO. The modification of the cross-sectional design, tip taper, and heat-treatment of WOG resulted in greater cyclic fatigue resistance in comparison with WO [11,12]. However, the new files are still used in the same reciprocating motion as WO, i.e., 150-degree counterclockwise and 30-degree clockwise [18,19]. Both the properties of the NiTi alloy and the cross-sectional design could affect the mechanical performance of the instrument, especially the maximum distortion angle and the TLP [20,21]. So far, there are no published reports on whether the dedicated reciprocating motion for WO offers safe angular movement for the new WOG instruments. The aim of this study was to investigate the torsional limit before plastic deformation (TLP) and torsional resistance of WOG instruments compared to WO instruments.

## 2. Materials and Methods

**Figure 1:**
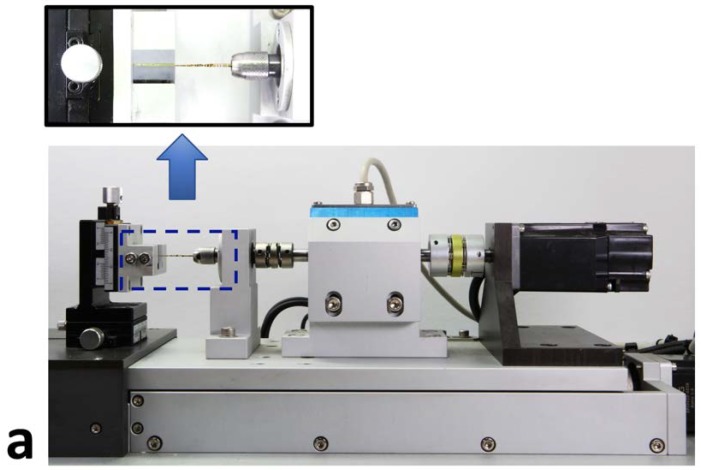

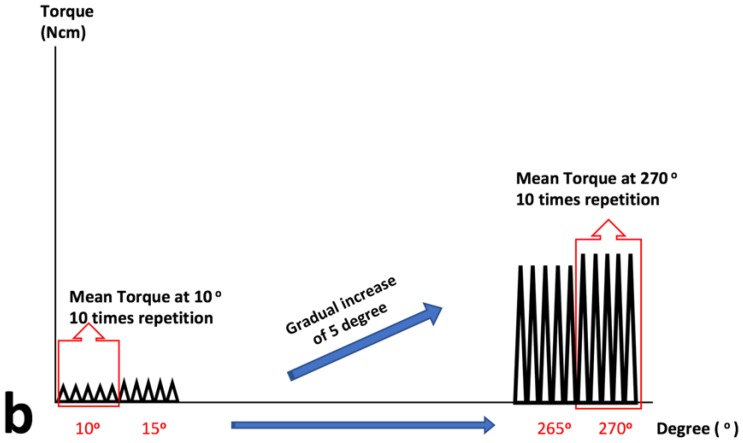
Custom-designed device used in this study. (**a**) File holding part (dotted box, magnified view) and (**b**) method to make repetitive torsional load conditions by automatic repetition of rotational load using gradual increase of rotational degree.

WO Primary and WOG Primary were tested in this study. Using a custom-designed device (Figure 1), repetitive torsional load conditions were applied by programming the specific rotational movements and resulting output variables into the control system [19].

### 2.1. Repetitive Continuous Torsional Test

The tested files were securely clamped between two brass plates at 3 mm from their tip. Repetitive torsional loads from reciprocating movements (counterclockwise/clockwise) were applied at 2 rpm with gradually increasing rotational angles. The repetitive torsional loads were applied from 10-degree to 270-degree rotation. The increments were in 5-degree intervals, where each interval consisted of 5 repetitive loading movements. Rotational movement (degree) and torsional load (Ncm) were recorded during the loading at a sampling rate of 20 Hz. A 50 milliseconds dwell time was programmed between rotation direction reversals. Sample size was 15 per group.

Mean maximum torsional load during the 5 repetitive reciprocating movements was computed at every 5-degree interval. The results were plotted in a rotational angle-torque graph with Origin 6.0 (Microcal Software Inc., Northampton, MA, USA). From this graph, the rotational angle and torsion load at the TLP were determined where the curve flattened out to plateau and stress-induced martensitic deformation happens.

### 2.2. Repetitive and Single Continuous Torsional Test

**Figure 2:**
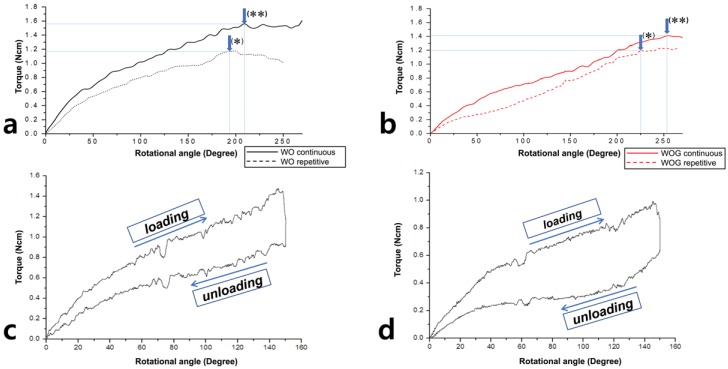
Representative superimposed plot acquired from the repetitive (*dotted line*) and single continuous rotation (*solid line*) torsional test for (**a**) WO and *(***b**) WOG. *Arrows* indicate the TLP from the (**a**) repetitive and (**b**) continuous rotation torsional test. A typical strip chart for (**c**) WO and (**d**) WOG showed that single continuous rotation to 150-degree and returning to original position result in superelastic response.

Another 15 files of each group were rotated in a single continuous movement until failure using counterclockwise (active cutting direction) rotation at a constant 2 rpm. The TLP were determined from the plotted load-distortion chart (Origin 6.0), and were compared with the results from the repetitive loading test (Figure 2a,b).

### 2.3. Single Continuous Rotation to 150-Degree and Returning to Original Position

To confirm the 150-degree angle set at the dedicated motor as a safety margin, the instruments were rotated up to 150-degrees and immediately returned to original position at a constant 2 rpm. Acquired data at 1000 Hz was plotted (Figure 2c,d). Sample size was 15 for each file system.

### 2.4. Statistics and Scanning Electron Microscope (SEM) Evaluation

After confirming normal data distributions using the Kolmogorov-Smirnov normality test, the data were statistically analyzed using an independent t-test at a confidence level of 95% (SPSS v 23.0 for Mac; IBM Corp., Somers, NY, USA). Based on a preliminary result, a prior sample size calculation was performed for the torsional resistance data using G*Power 3.1 software for Mac (Heinrich Heine University Düsseldorf, Düsseldorf, Germany) [22]. Alpha-type error was set at 0.05 and beta power at 0.80, and a N2/N1 ratio of 1 was established. Nine for WO and 15 for WOG were determined. Therefore, the sample size was proved to be proper by a prior sample size calculation. After continuous and repetitive torsional tests, five specimens were selected randomly for observation under a SEM (SU8230; Hitachi High-Technologies Corporation, Tokyo, Japan) to evaluate the surface features of the loaded area and topographic features of the fractured surfaces.

## 3. Results

Table 1 summarizes the torsional resistance parameters of WO and WOG. 

Regardless of the test modes, at the TLP for the 3-mm tip restriction level, the torsional loads were not significantly different between WO and WOG. The distortion angle of WOG was however significantly higher than WO (*p* < 0.05). Regardless of the instrument type, the distortional angle at TLP was greater than the 150-degree (Table 1). Meanwhile, torsional loads within each loading type (continuous or repetitive) were not significantly different between WO and WOG instruments (*p* > 0.05).

Repetitive loading movements significantly decreased the distortion angle and torsional load in WO and WOG instruments when compared to continuous movement (*p* < 0.05) (Figure 2a,b). A plot of single continuous rotation to 150-degrees and return showed an elastic response by the absence of plastic deformation (Figure 2c,d).

**Figure 3:**
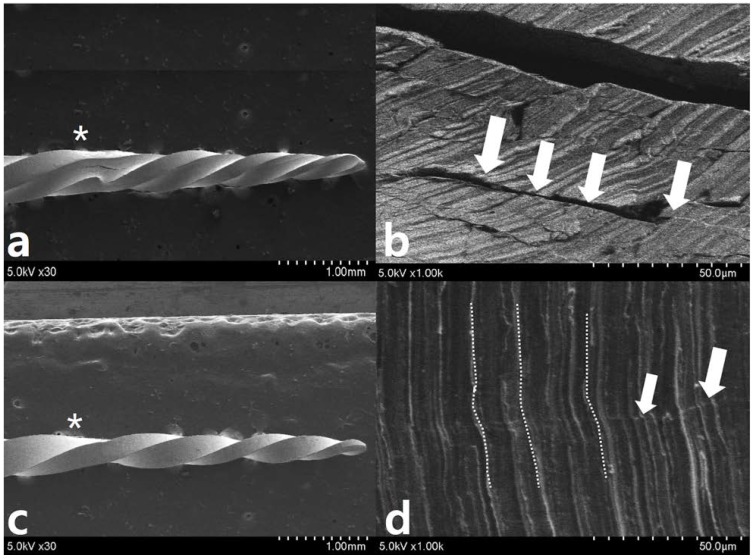
Scanning electron microscopy observation after 270-degree repetitive loading for (**a**,**b**) WO and (**c**,**d**) WOG (* indicates the restricted area of 3 mm level). SEM evaluation of the lateral aspects shows longitudinal micro-cracks (*white arrows* in (**b**,**d**)) running along the long axis of the file shaft around 3 mm from tip (*). While (**a**,**b**) WO shows catastrophic change like a fault after an earthquake, WOG shows distorted machining grooves (*dotted lines* in (**d**)) and a few longitudinal micro-cracks (*white arrows* in (**d**)).

**Figure 4:**
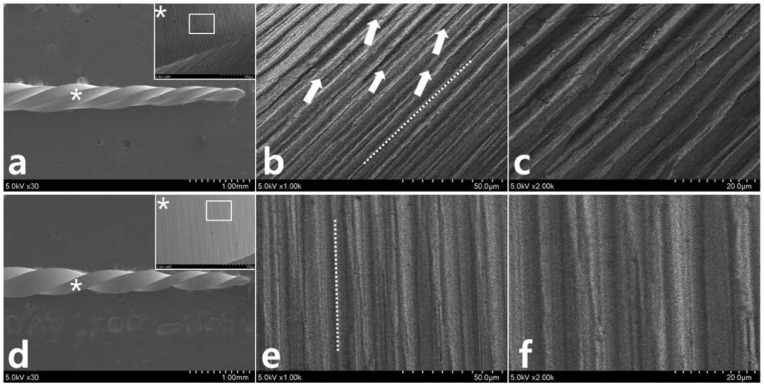
Scanning electron microscopy observation after 150-degree repetitive loading for (**a**) WO and (**d**) WOG (* indicates the restricted area of 3 mm level). (**b**) WO and (**e**) WOG show machining grooves (dotted lines) without distortion (magnified views (**b**,**e**) are from the box in the (**a**,**d**), respectively). WO shows evidence of longitudinal micro-cracks (*white arrows* in (**b**)). (**c**,**f**) are the magnified aspects from (**b**,**e**), respectively.

**Figure 5:**
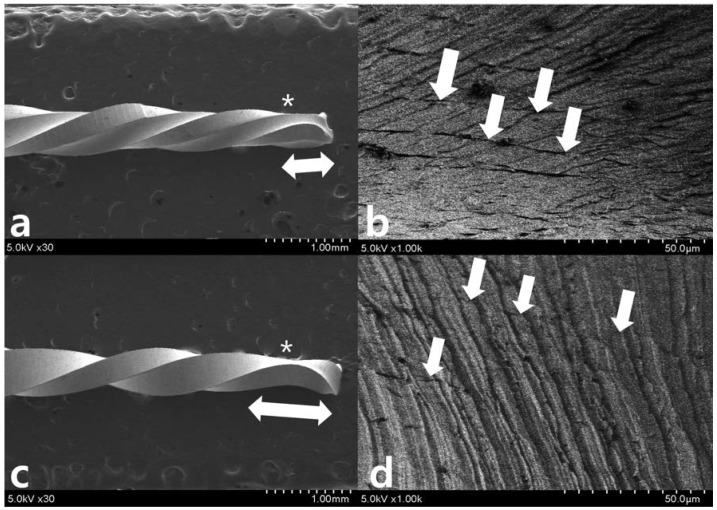
Scanning electron microscopy images after single continuous rotation test, WO (**a**,**b**) revealed much more evidence of micro-cracks (*white arrows*) than the WOG (**c**,**d**). The lateral aspect of fractured specimens showed not only numerous longitudinal micro-cracks but also distortion of the flute. The range of flute distortion (*double-headed arrows*) of WOG (**c**) was longer than that of WO (**a**).

**Figure 6:**
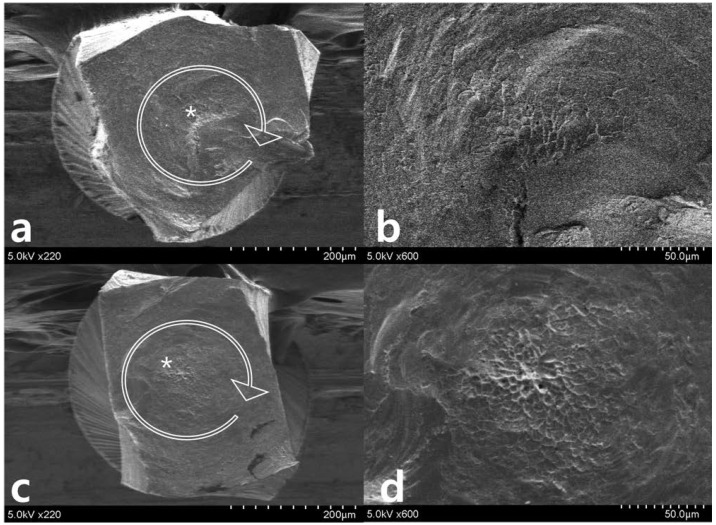
Scanning electron microscopy images of the fractured cross-sectional surfaces after single continuous rotation test, (**a**,**b**) WO and (**c**,**d**) WOG. The cross-sectional images revealed typical features of torsional fractures, concentric abrasion marks (*circle arrows* in (**a**,**c**)), and fibrous dimples from the torsional center (**b**,**d**).

After repetitive movement with an incrementally increasing loading angle up to 270 degrees (Figure 3), SEM evaluation of the lateral aspects showed longitudinal micro-cracks running along the long axis of the file shaft (Figure 3a–d). While WO (Figure 3a,b) begins to fracture partially, WOG (Figure 3c,d) shows distorted machining grooves (white dotted line in Figure 3d) and a few longitudinal cracks (white arrow in Figure 3d). After repetitive movement with an incremental increase in loading angle up to 150 degrees (Figure 4), no distorted machining grooves were found in either WO or WOG groups (see white dotted line), but WO showed evidence of longitudinal micro-cracks (white arrows) (Figure 4b,c). After the single continuous rotation test (Figure 5), WO revealed much more evidence of micro-cracks (white arrows) than the WOG (Figure 5b,d). The lateral aspect of fractured specimens showed not only numerous longitudinal micro-cracks but also distortion of the flute. The length of unwound distortion of WOG was longer than that of WO (Figure 5a,c). The fractured cross-sectional surfaces from both groups revealed typical features of torsional fractures, such as concentric abrasion marks and fibrous dimples from the torsional center (Figure 6).

## 4. Discussion

Manufacturers claim that reciprocating mechanisms improve cyclic fatigue resistance and avoid torsional failure by reversing the direction of rotation for short intermittent segments, resulting in less movement in the cutting direction [23]. Previous reports support such an increase in cyclic fatigue resistance by reciprocating movements [13,18,19,23,24]. It has also been reported that the pre-set rotation angle of dedicated motors for the reciprocating systems is less than the rotation that causes plastic deformation and less than the TLP [18,19].

However, the pre-set angle rotation of dedicated motors was originally designed for Reciproc and WO made from M-wire, while Reciproc Blue and WOG are made from an NiTi alloy types with different mechanical properties [21,25]. Whether the original motor pre-sets of the reciprocation angle remains within the safe TLP limit for the new instruments still needs to be confirmed.

The results from the two tests done in this study indicate that the TLP of WOG had a higher rotation angle than the 150-degree rotation in the WO dedicated motors. This was consistent with previous studies that reported that the TLP of Reciproc and WO were higher than 150-degrees [18,19]. Hence, the 150-degree counterclockwise rotation applied by the dedicated motors is within the safe rotational degree of the WOG instrument. The SEM observation of WOG after gradually increasing repetitive angular movements up to 150-degrees did also not show any topographic change in the WOG. In general, instruments with small cross-sectional areas reportedly have lower torsional resistance than larger ones [26]. In this study, the instrument’s tip was secured at 3 mm level so it is possible that the distortion angle and torsional load might be lower and closer to the file tip.

In a previous study, it was shown that the resulting torsional load generated from gradually increasing repetitive angular movements reached the TLP at a lower rotational angle and torsional load than those in a single continuous rotational movement [19]. This difference indicated that a repetitive loading movement, even when it is within the original TLP, may reduce the maximum torsional load and distortion angle of an instrument. The SEM images of WO after 150-degree repetitive loading movement showed some longitudinal micro-cracks (Figure 4b,c). It is possible that repetitive reciprocating tensile and compressive deformation, along the long axis of an instrument, accumulate local damage and thereby reach the TLP. This could pose a risk for torsional fractures if the file is reused for multiple canals or cases. 

In the SEM evaluation, the lateral aspect of most groups showed numerous longitudinal micro-cracks, which is suspected to dislocations, running along the long axis of the file shaft. This was more prominent for WO than WOG groups. Meanwhile, distorted (not straight) machining grooves could be observed clearly for WOG. After repetitive loading movements, WOG did not show any topographic change under SEM observation up to ×10,000 magnification. Meanwhile, SEM observation supports that repeated reciprocating motion caused catastrophic change on the file’s lateral surface without either fracture or plastic deformation evidence (Figure 3). Localized permanent deformation or distortion may result in reduced clinical efficiency for root canal preparation and debris retrieval and increased risk of instrument breakage.

## 5. Conclusions

Based on the present study, the WOG reciprocating file was found to have a safe rotational angle for the cutting movement when used in a motor dedicated to WO. However, clinicians need to consider that decreased TLP of a root canal instrument by repetitive torsional loading and/or reuse could result in a higher chance of permanent deformation or distortion when files bind within a root canal. 

Therefore, the results of this study support that the dedicated motor for WO does not exceed the TLP of the WOG in the pre-set mode if a reciprocating file is used for a restricted time (single case use). Further studies will be needed to investigate other reciprocating angles or kinetics under varying conditions, and the potential reduction in the TLP.

## Figures and Tables

**Table 1:** Maximum distortion angle (degree) and torsional load (Ncm) at the torsional limit before plastic deformation (TLP) (mean ± SD), depending on test condition.

	Distortion Angle (°)		Torsional Load (Ncm)	
	WaveOne	WaveOne Gold	WaveOne	WaveOne Gold
Continuous	209 ± 16 *^,†^	243 ± 20 *^,†^	1.49 ± 0.25 ^†^	1.39 ± 0.24 ^†^
Repetitive	190 ± 9 *^,^^†^	220 ± 15 *^,^^†^	1.13 ± 0.11 ^†^	1.20 ± 0.12 ^†^

* Asterisk superscript symbol indicates significant difference between WaveOne and WaveOne Gold groups (*p* < 0.05). ^†^ Obelisk superscript symbol indicates significant difference between continuous and repetitive test modes (*p* < 0.05).
